# An Innovative Wearable Device For Monitoring Continuous Body Surface Temperature (HEARThermo): Instrument Validation Study

**DOI:** 10.2196/19210

**Published:** 2021-02-10

**Authors:** Chun-Yin Yeh, Yi-Ting Chung, Kun-Ta Chuang, Yu-Chen Shu, Hung-Yu Kao, Po-Lin Chen, Wen-Chien Ko, Nai-Ying Ko

**Affiliations:** 1 Department of Computer Science and Information Engineering National Cheng Kung University Tainan Taiwan; 2 Department of Nursing National Cheng Kung University Tainan Taiwan; 3 Department of Mathematics National Cheng Kung University Tainan Taiwan; 4 Department of Medicine National Cheng Kung University Tainan Taiwan; 5 Department of Microbiology and Immunology National Cheng Kung University Tainan Taiwan; 6 Department of Internal Medicine National Cheng Kung University Hospital Tainan Taiwan; 7 Department of Public Health National Cheng Kung University Tainan Taiwan

**Keywords:** body surface temperature, wearable device, validation, continuous monitoring

## Abstract

**Background:**

Variations in body temperature are highly informative during an illness. To date, there are not many adequate studies that have investigated the feasibility of a wearable wrist device for the continuous monitoring of body surface temperatures in humans.

**Objective:**

The objective of this study was to validate the performance of HEARThermo, an innovative wearable device, which was developed to continuously monitor the body surface temperature in humans.

**Methods:**

We implemented a multi-method research design in this study, which included 2 validation studies—one in the laboratory and one with human subjects. In validation study I, we evaluated the test-retest reliability of HEARThermo in the laboratory to measure the temperature and to correct the values recorded by each HEARThermo by using linear regression models. We conducted validation study II on human subjects who wore HEARThermo for the measurement of their body surface temperatures. Additionally, we compared the HEARThermo temperature recordings with those recorded by the infrared skin thermometer simultaneously. We used intraclass correlation coefficients (ICCs) and Bland-Altman plots to analyze the criterion validity and agreement between the 2 measurement tools.

**Results:**

A total of 66 participants (age range, 10-77 years) were recruited, and 152,881 completed data were analyzed in this study. The 2 validation studies in the laboratory and on human skin indicated that HEARThermo showed a good test-retest reliability (ICC 0.96-0.98) and adequate criterion validity with the infrared skin thermometer at room temperatures of 20°C-27.9°C (ICC 0.72, *P*<.001). The corrected measurement bias averaged –0.02°C, which was calibrated using a water bath ranging in temperature from 16°C to 40°C. The values of each HEARThermo improved by the regression models were not significantly different from the temperature of the water bath (*P*=.19). Bland-Altman plots showed no visualized systematic bias. HEARThermo had a bias of 1.51°C with a 95% limit of agreement between –1.34°C and 4.35°C.

**Conclusions:**

The findings of our study show the validation of HEARThermo for the continuous monitoring of body surface temperatures in humans.

## Introduction

### Background

Abnormalities in body temperature are the key indicators for the prognosis of various illnesses [1-4] and the most common symptoms of defensive immune responses [4]. Abnormal body temperature patterns are informative and specific to poor progress during an illness, regardless of whether people meet the criteria for fever or not [5-10]. Large body temperature variations before the development of a fever indicate the evolution of sepsis syndrome [9]. A retrospective study found that afebrile critically ill patients with abnormal temperature patterns in any 24-hour period had a 4.43-fold risk for subsequent diagnosis of sepsis as compared to patients without such patterns, and the sensitivity and specificity of the abnormal temperature curves to predict sepsis were 0.69 and 0.76, respectively [8]. At the individual level, an unexplained variation in 1 SD increase in body temperature among outpatients is a significant predictor linked to 8.4% higher rate of 1-year mortality [11]. Therefore, body temperature is one of the most important vital signs for evaluating human health, and continuous monitoring should be done for the early identification of patients who are at a risk for poor prognosis [12,13].

Thermometers such as glass mercury thermometers, electronic thermometers, infrared ear thermometers, and infrared forehead thermometers are used to measure the human body temperature. They are usually used at predetermined intervals or when a patient’s condition changes [7,14]. Considering the equivalents of intravascular or direct brain thermometry, the measurement tools for continuous body temperature monitoring in clinical practice are invasive and include urinary catheters or esophageal temperature probes [15]. However, such invasive continuous body temperature measurement tools might not be applicable to the general population.

To date, wearable devices with the advantage of minimizing discomfort and interference with normal human activities have garnered increased attention in the field of continuous monitoring of body temperatures [13,16,17]. Different wearable devices, including skin-like arrays of precision temperature sensors or wearable adhesive devices such as watches, vest, patches, and earphones, have been developed to continuously examine the body temperature [18-21]. Among the human body parts, the wrist is the most responsive body part for wearable devices that measure thermal sensation [19,22]. However, the applications of wearable devices in the continuous monitoring of body temperature are still limited in terms of pilot studies [18,20,23]. In addition, the validity of wrist skin temperature monitoring using novel wearable devices in both laboratory settings and on human subjects has not been sufficiently investigated.

### Prior Work and Objective of This Study

An early study conducted in 2006 was the first study to use iButton, which has a mean accuracy of 0.05°C through a validation study by using a water bath with different temperatures and a reference thermometer. This study further underscored the generalizability of laboratory findings to clinical settings [24]. Most studies were conducted to assess the feasibility of the wrist skin temperature measured by iButtons in terms of the human circadian system [25-27]. Two studies conducted on approximately 100 university students in Spain proved that the wrist temperature rhythm is a valuable index for assessing the circadian rhythm [25,26]. Another study conducted on 121 shift workers in Korea provided evidence that lower wrist temperature amplitudes appeared more in the nightshift workers than in the dayshift workers [27]. In addition, several studies were conducted to assess the effects of continuously monitoring body temperature by using wearable devices for the detection of worsening conditions in patients [6,10,23,28,29]. A prospective observational study consisting of 56 patients was carried out to explore the effects of a Holter device with both central and peripheral infrared temperatures being recorded and stored every minute for 24 hours. It was found that 0.7 peaks of fever per patient could be detected by continuous monitoring but this was unobserved during conventional care, and 16% of the patients considered afebrile by conventional care had at least one fever peak detected during continuous monitoring [29]. Another pilot study of pediatric patients indicated that 2 fever events could not be detected by routine temperature monitoring but they could be detected 12 hours earlier by a continuous temperature monitoring patch [23]. However, the limitations of these devices included a long response time due to their relatively large sizes, and the maximum sampling rate was 1 per minute [24]. Moreover, these studies did not show the reliability and validity of the wearable devices in terms of monitoring body surface temperature [25-27]. Therefore, the objective of this study was to validate the performance of HEARThermo, an innovative wearable device, which was developed to continuously monitor the body temperature in humans.

## Methods

### Study Design

We used a multi-method research design in this study, which included 2 validation studies—one in the laboratory and one with human subjects. We conducted these studies to determine the reliability of HEARThermo in the laboratory setting and the agreement and validity of HEARThermo for application on human skin. The Institutional Review Board at the National Cheng Kung University Hospital (no. B-BR-106-044) approved this study’s protocol.

### Study Participants

Snowball sampling was conducted to recruit people who were able to communicate in Taiwanese or Mandarin, who lived in the Tainan community, and who were willing to participate in this study. The exclusion criteria included people (1) with fever or if they experienced physical discomfort 3 days before participation in this study; (2) with severe brain injury, neurological disease, severe cardiovascular disease, ear structure problem, peripheral artery disease, or musculoskeletal disease in the limbs; (3) with mental disorders or cognitive disability; and (4) under medication with effects on the vital markers such as corticosteroids, nonsteroidal anti-inflammatory drugs, or anti-fever medications 4 hours before the commencement of this study.

We recruited 66 participants in this study and all of them completed the experimental study. The power analysis was calculated using “ICC.Sample.Size” packages by R Version 3.4.1 (R Statistics Software). The post hoc power analysis indicated a sample size of 66 participants to detect a high-power level (power=0.99).

### Data Collection

#### Validation Study I

The methods of validating HEARThermo in the laboratory setting used in this study were conducted in accordance with Section 4.5.1 “Operating Environment Tests” cited in the “National Standards of the Republic of China (CNS) 15043 Standard specification for electronic thermometer for intermittent determination of patient temperatures” [30]. According to the instructions for wearable devices, they were stabilized for 1 hour before the validation tests. We tested HEARThermo in a water bath at 16°C and 40°C thrice and recorded the measurement error range. We verified the temperature of the water bath with the reference mercury thermometer.

#### Validation Study II

**Figure 1:**
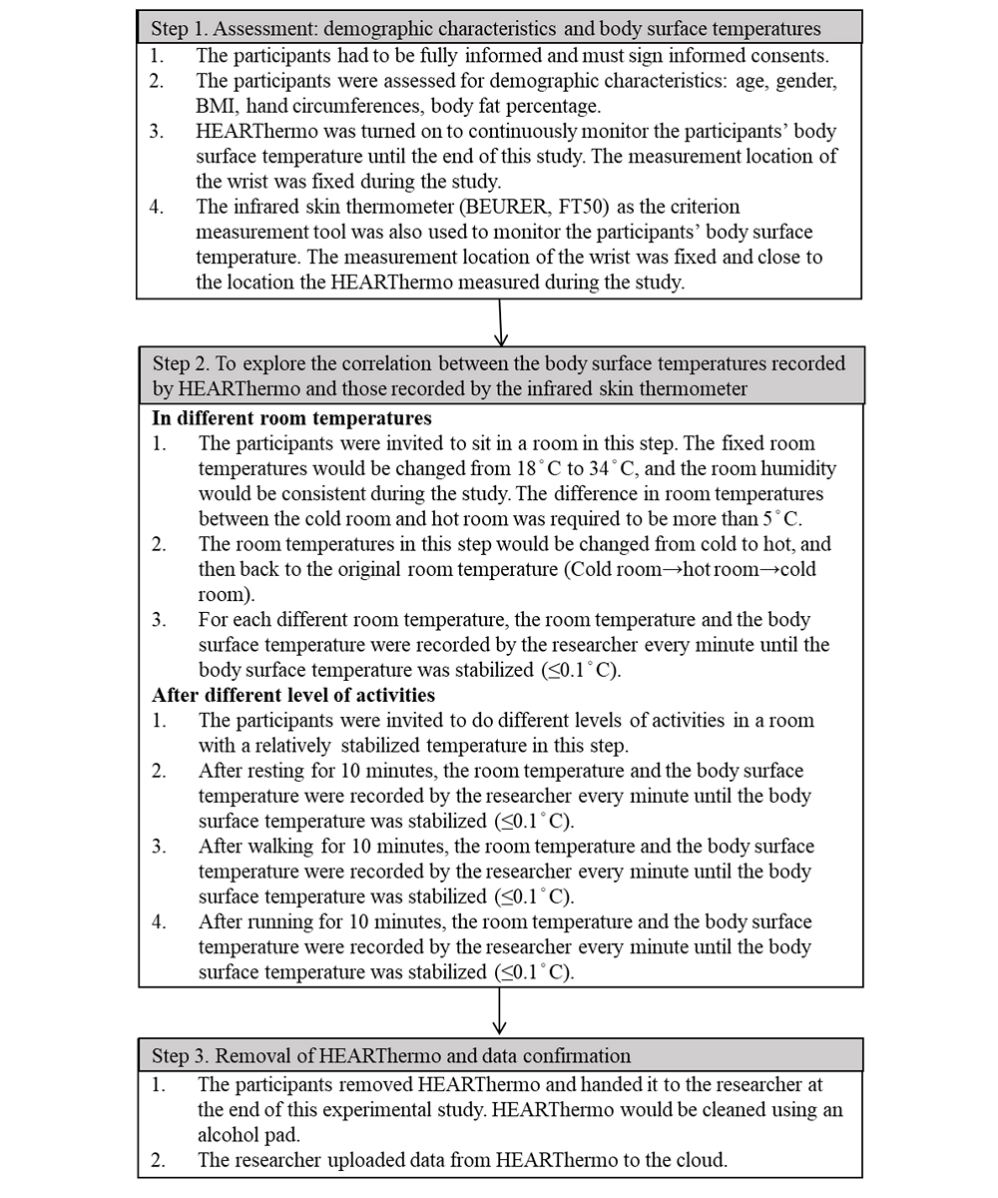
The protocol for validation study II.

We conducted a validation study II to determine the correlation between the body surface temperature obtained by HEARThermo and that obtained by the infrared skin thermometer. We used HEARThermo to continuously monitor the participants’ body surface temperatures until the end of the study. The time to participate in the study was between 9 AM and 11 AM, and the rooms for the study were fixed to avoid any potential bias from the environment. Since the emissivity of infrared thermometers might be affected by environmental temperature [31], the different room temperatures, humidity, and activity levels were taken into consideration as confounders in the validation study. Figure 1 shows the details of the validation study II protocol.

### Variable Measurements

#### Demographic Characteristics

The individual demographic data collected for this study included age, gender, height, weight, BMI, body fat percentage, and hand circumference.

#### Body Surface Temperature

We used an infrared skin thermometer (BEURER, FT50) to measure the body surface temperature in the experimental study. The researcher first cleaned specific places on the monitored hands, and after the skin was dry, an infrared skin thermometer was placed on the skin surface of the hands. The measured temperature appeared on the display along with a face symbol after the SCAN button was released. According to the instructions for the infrared skin thermometer used in this study, the measurement error range is ±0.3°C at temperatures from 10°C to 50°C and ±0.1°C at temperatures from 37°C to 39°C [32]. The sampling frequency of the infrared skin thermometer is 512 scanning sequences per second [32]. The accuracy test of the infrared skin thermometer used in this study was conducted in accordance with Section 5.1.5 “Operating Environment Tests” cited in the “CNS 15042 Standard specification for infrared thermometer for intermittent determination of patient temperatures” [31]. First, the infrared skin thermometer was stabilized for 30 minutes before the accuracy tests. The infrared skin thermometer was then tested 6 times in a water bath at 23°C, 30°C, and 38°C, and the measurement error range was recorded. The results revealed that the infrared skin thermometer meets the acceptance criteria of the CNS standards, for which the maximum measurement error is required to be ±0.3°C.

**Figure 2:**
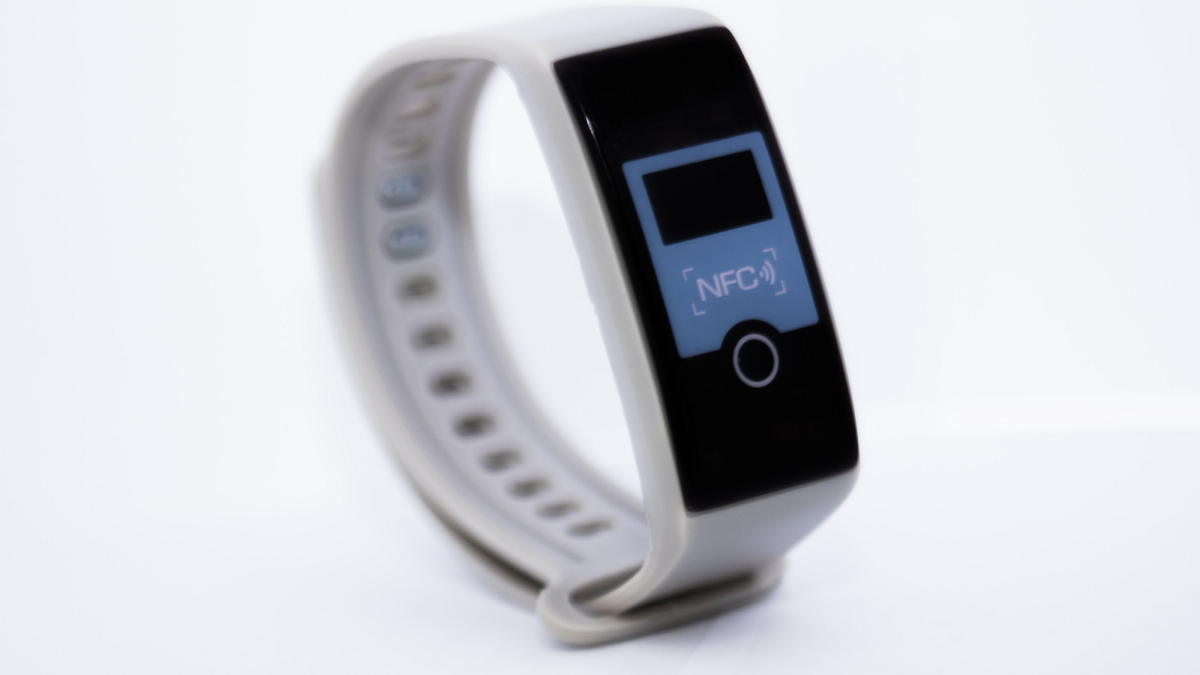
HEARThermo device.

HEARThermo, which was developed in cooperation with the technology manufacturer (AMobile Intelligent Corp Ltd), was used to continuously measure the body surface temperature in the validation study II (Figure 2). In contrast to the previously developed devices with thermistors and integrated circuits sensors [23,24,29,33,34], this wearable device comprises the TEMPUS Digital Far Infrared Thermopile Sensor to measure the far infrared energy emitted from the body surface and presents the temperatures immediately. The signal measurement rate of HEARThermo is >500 Hz. Besides, the sensors of HEARThermo also include g-sensor, gyroscope, heart rate, and 3D accelerometer. In this study, the researcher first cleaned specific places on the monitored hands, and after the skin was dry, HEARThermo was lightly placed on the skin surface of the wrist. When HEARThermo was turned on, the measured parameters, including temperature and heart rate, appeared on the display every second. These physiological biomarkers were uploaded through the Bluetooth Low Energy gateway to the National Cheng Kung University cloud.

#### Room Temperature and Humidity

We used a room temperature data logger (Elitech, RC-4) to measure the room temperature in this study. According to the manufacturer’s instructions, the temperature accuracy was ±0.5°C at temperatures from –20°C to 40°C [35]. A room humidity data logger (N Dr.AV, GM-108) was used to measure the room humidity. According to the manufacturer’s instructions, the humidity accuracy is ±10% for humidity ranging from 20%-90% [36].

### Data Analysis

We examined the differences between the groups by using analysis of variance and chi-square test for the categorical variables. Then, we calculated the measurement bias of each HEARThermo as the mean (SD) of the differences between the temperatures of HEARThermo and that of the water bath. Moreover, we calculated the linear regressions between the temperatures of the water bath and each HEARThermo to determine the relationship between the values of the wearable devices and those of the water bath. We applied the linear regression model for each HEARThermo to an independent data set to correct the values of each wearable device and tested the improvements in the values with a two-tailed paired *t* test by using the biases of the absolute values. To determine the test-retest reliability of the individual wearable device, we analyzed the intraclass correlation coefficient (ICC) of each HEARThermo. We analyzed the agreement between HEARThermo and the infrared skin thermometer by using Bland-Altman plots. Additionally, we calculated the means, namely bias, and SD, namely precision, of the differences, and 95% limits of agreement. Furthermore, we analyzed the criterion validity of the HEARThermo by using ICC two-way random effect model with 95% CIs. We conducted all the statistical analyses by using R Version 3.4.1.

## Results

### Study Population

We included 66 participants in Tainan City, Taiwan for this experimental study (Multimedia Appendix 1). A total of 325,022 data were observed, and 152,881 completed data were analyzed. The age of the participants ranged from 10 years to 77 years, with a mean (SD) age of 39.47 (19.02) years, and 39 out of the 66 participants (59%) were female. The mean (SD) height of these participants was 161.4 (10.85) cm, the mean (SD) weight was 60.82 (14.83) kg, the mean (SD) BMI was 23 (3.41), the mean (SD) body fat percentage was 26.61 (6.98)%, and the mean (SD) hand circumference was 15.38 (1.61) cm. The mean (SD) stabilized time after walking was 357.06 (192.24) seconds, and the mean (SD) stabilized time after running was 790.67 (259.69) seconds.

### Validation Study I: Reliability of HEARThermo in the Laboratory Setting

We tested 39 wearable devices thrice in a water bath at 16°C and 40°C. The mean (SD) measurement bias at 16°C amounted to −1.16 (1.84)°C (range –4.91°C to 2.18°C). The mean (SD) measurement bias at 40°C amounted to 1.21 (2.01)°C (range –2.39°C to 5.63°C). The ICC for the water bath at 16°C was 0.96, whereas the ICC for the water bath at 40°C was 0.98 (Table 1).

**Table 1:** Distributions of the temperatures of the 39 HEARThermo devices in the water bath.

Temperature of the water bath	Tests (n)	Minimum (°C)	Median (Q1-Q3)	Maximum (°C)	Mean (SD)	CV^a^	ICC^b^
16°C	117	11.09	14.94 (13.3-16.49)	18.18	14.84 (1.84)	12.38	0.96
40°C	117	37.61	41.04 (39.72-42.8)	45.63	41.21 (2.01)	4.876	0.98

^a^CV: coefficient of variance.

^b^ICC: intraclass correlation coefficient.

We conducted linear regressions to calibrate each wearable device. The intercept calibration coefficients ranged from –3.20 to 7.07°C and the slope calibration coefficients ranged from 0.77 to 1.11°C. The corrected measurement bias averaged –0.02 (SD 0.28)°C (range –1.79°C to 0.81°C). The values of each HEARThermo improved by the regression models were not significantly different from the temperatures of the water bath (*P*=.19).

### Validation Study II: Agreement and Validity of HEARThermo for Application on Humans

**Figure 3:**
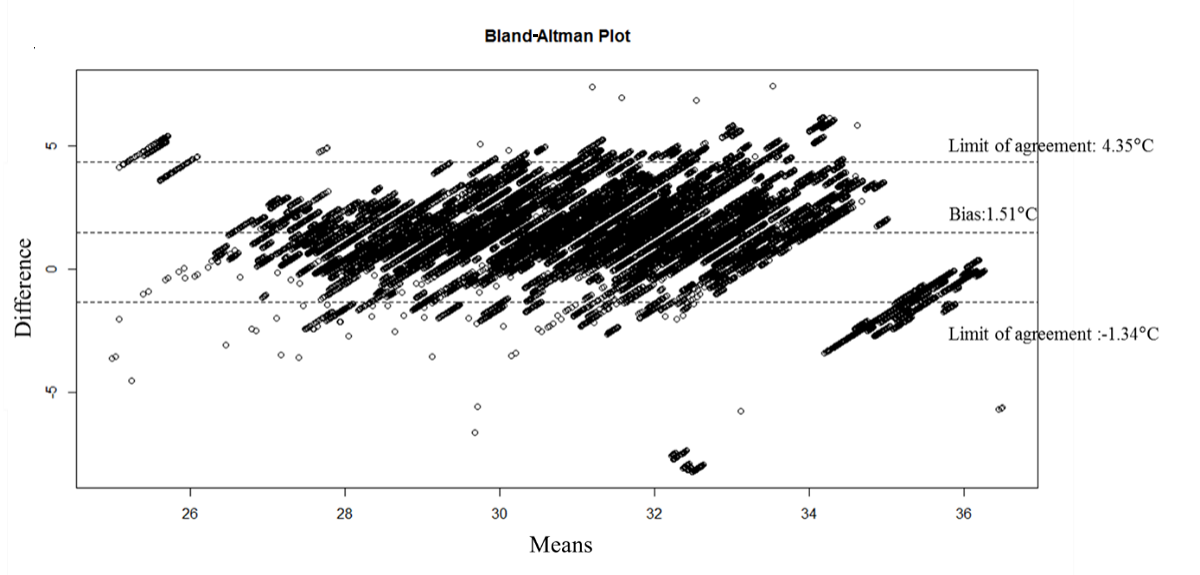
Bland-Altman plots of level of agreement between HEARThermo and the infrared skin thermometers.

Figure 3 shows the level of agreement of body surface temperatures between HEARThermo and the infrared skin thermometer. The mean (SD) temperature of HEARThermo was 31.94 (2.04)°C as compared with 30.43 (2.02)°C for the infrared skin thermometer. Bland-Altman analyses indicated that the bias of HEARThermo was 1.51°C (95% CI 1.50-1.51) with a precision of 1.45. The limit of agreement was –1.34°C (95% CI –1.35 to –1.32) to 4.35°C (95% CI 4.34-4.36). Table 2 shows the ICC values between the wearable devices and the infrared skin thermometer at different room temperatures, which included ≤19.9°C, 20°C-27.9°C, and ≥28°C. The ICC was high with an estimate of 0.72 for the room temperatures of 20°C-27.9°C. At room temperatures of 20°C-27.9°C, the mean (SD) temperature of HEARThermo was 32.44 (1.67)°C (range 25.45°C-37.53°C) compared with 30.86 (1.85)°C (range 25.7°C-36.6°C) for the infrared skin thermometer. The ICC values declined rapidly with variations in room temperature with low ICC values of 0.45 and 0.49. At room temperature ≤19.9°C, the mean (SD) temperature of HEARThermo was 28.41 (0.92)°C (range 24.07°C-29.81°C) compared with 26.72 (2.05)°C (range 23.0°C-29.4°C) for the infrared skin thermometer. At room temperatures ≥28°C, the temperature variation of HEARThermo was 34.17 (1.22)°C (range 26.93°C-36.49°C) compared with 33.42 (1.78)°C (range 30.2°C-36.6°C) for the infrared skin thermometer.

**Table 2:** Intraclass correlation coefficients between HEARThermo and infrared skin thermometer.

Room temperature (°C)	Intraclass correlation coefficient	95% CI	*F* value *(df)*	*P* value
≤19.9	0.45	0.40-0.49	2.62 (1354)	<.001
20-27.9	0.72	0.71-0.72	6.04 (140292)	<.001
≥28	0.49	0.48-0.51	2.94 (11233)	<.001

## Discussion

### Overview of the Findings

This is the first study to investigate the reliability and validity of novel wearable wrist devices with thermopiles for continuous monitoring of body surface temperature in a laboratory setting and on human subjects. Our study findings indicated that HEARThermo shows good reliability and adequate validity with infrared skin thermometers. The validated HEARThermo could be expected to provide more useful information by continuously monitoring variations in the body surface temperatures to support medical decisions more effectively. HEARThermo is a reliable device with a high ICC of 0.96-0.98 for the repeated measurement of body surface temperatures, which is consistent with that reported in a previous comparative study conducted on humans [37]. A recent systematic review of the measurement of body surface temperatures using contact thermometry showed that 94% of the studies lack detailed information about sensor calibration, thereby resulting in reduced validity of the data [38]. In this study, HEARThermo devices were calibrated using a water bath, and the corrected measurement bias averaged –0.02°C, which was smaller than that of the previously developed devices with ranges from 0.05°C to 0.2°C [23,24,29,33]. Measurement bias less than 0.5°C is considered minor [38]. However, the correction models should be adjusted occasionally because of the complex process of validating results due to the high demands on the specifications of the calibration methods and the reference thermometer [24]. Therefore, we suggest that the validation and calibration process for an innovative device should be based on the purposes of application in monitoring body surface temperatures under different scenarios.

HEARThermo was sufficiently validated with the infrared skin thermometer at room temperatures ranging from 20°C to 27.9°C, which concurred with the comparative study between iButton and reference mercury thermometer in a temperature-controlled water bath [39]. Studies have shown that the most used sensors in wearable wrist devices for monitoring body surface temperatures are thermistors [21,34,38], but only limited studies have tried to validate these wearable devices by using a temperature-controlled water bath from 19°C to 41°C [19,24,40]. Our study is the first to provide supporting evidence for the validity of infrared wearable devices in real time for measuring body temperatures in humans. Furthermore, HEARThermo showed no visualized systematic bias in terms of the criterion for infrared skin thermometers. However, the limits of agreement between the 2 measurement tools were wider than was the case in previous studies, which suggested that the range for the adequate agreement should be within 1°C in in vivo studies [38] and 0.5°C in clinical practice [41]. The effects of external factors on changing emissivity of the body surface may be more significant for infrared skin thermometers than for wearable devices due to the intermittent measurements by infrared skin thermometers [19,42], which may account for the wider range of limits of agreement found in this study. Our results reinforce that measuring body surface temperature using wearable devices should consider the tight contact with the skin surface. To date, wearable devices in the form of patches to monitor body temperature with features of tight contact have been developed and approved by the US Food and Drug Administration [23]. However, the ability to compare the agreement among different measurement tools for body surface temperatures might be limited due to the lack of a universal gold standard method for measuring body surface temperatures [38]. Further studies are needed to calibrate and validate the agreement between HEARThermo and different forms of medical thermometers in clinical settings.

### Limitations

This study has the following limitations. First, the sensors of HEARThermo could not be calibrated using a high-precision blackbody while controlling the source of thermal radiation in this study. Second, a gold standard for measuring body surface temperatures is lacking, which might cause validation results to vary from one criterion measurement tool to another. Therefore, our study only highlights the relationship between HEARThermo and the infrared skin thermometer used in this study. Third, the generalizability of the study findings was limited to people aged ≥10 years since there was no appropriate size of bracelet for children aged 0-9 years. In addition, relatively healthy people with greater willingness might be recruited because of the snowball sampling method. However, as compared to the previous studies with small sample sizes and only males, this study tried to recruit both females and males and involved a larger sample size and wider age range to improve the generalizability of the findings. Fourth, the results of this study would be more applicable in Asian countries as only Asian people were recruited in this study. We recommend that further studies be conducted in different countries and races to validate wearable devices with infrared sensors in consideration of the emissivity of different human skin tones.

### Conclusion

HEARThermo showed good test-retest reliability in the laboratory setting, with the highest correlation with the infrared skin thermometer at room temperatures of 20°C-27.9°C. Moreover, HEARThermo showed no visualized systematic bias and had a bias of 1.51°C with the criterion for infrared skin thermometers. This study validated an innovative wearable device for continuous monitoring of body surface temperatures. Further studies are needed to calibrate and validate the agreement between HEARThermo and different forms of medical thermometers in clinical practice.

## Appendix

Multimedia Appendix 1 Characteristics of the 66 study participants.

## Abbreviations

CNS: National Standards of the Republic of China
ICC: intraclass correlation coefficient
