# Supplementary material for: An Innovative Wearable Device For Monitoring Continuous Body Surface Temperature (HEARThermo): Instrument Validation Study
Source: JMIR Mhealth Uhealth. 2021 Feb 10;9(2):e19210. doi: 10.2196/19210 (PMC7904403; doi:10.2196/19210)
Supplement: Multimedia Appendix 1 [file mhealth_v9i2e19210_app1.docx]

Appendix 1: Characteristics of the 66 study participants.

| Variables | | Total (N=66) | Age 10-19 (n=13) | Age 20-29 (n=11) | Age 30-39 (n=13) | Age 40-49 (n=8) | Age 50-59 (n=8) | Age 60-69 (n=8) | Age≥70 (n=5) | *P* value |
| --- | --- | --- | --- | --- | --- | --- | --- | --- | --- | --- |
| Age (years), mean (SD) | | 39.47 (19.02) | 16.77 (2.89) | 24.55 (3.39) | 33.69 (3.2) | 42.88 (2.64) | 55.5 (2.67) | 65.38 (3.07) | 73.8 (2.39) | <.001 |
| Gender, female, n (%) | | 39 (59.1) | 7 (53.8) | 5 (45.5) | 7 (53.8) | 5 (62.5) | 7 (87.5) | 6 (75) | 2 (40) | .49 |
| Height (cm), mean (SD) | | 161.4 (10.85) | 157.46 (15.45) | 165.36 (11.34) | 163.42 (8.74) | 165.25 (9.35) | 160.63 (5.34) | 158.88 (11.56) | 156.8 (5.76) | .43 |
| Weight (kg), mean (SD) | | 60.82 (14.83) | 57.25 (23.22) | 64.36 (11.93) | 64.55 (15.19) | 66.63 (14.2) | 54.3 (4.95) | 59.5 (9.81) | 55.84 (6.08) | .48 |
| BMI, mean (SD) | | 23 (3.41) | 22.11 (5.22) | 23.39 (2.58) | 23.94 (3.95) | 24.1 (2.8) | 21.05 (1.77) | 23.48 (1.99) | 22.66 (1.2) | .47 |
| Fat (%), mean (SD) | | 26.61 (6.98) | 23.98 (8.57) | 25.6 (8.67) | 26.62 (6.93) | 27.43 (4.54) | 27.7 (5.74) | 29.99 (6.71) | 27.22 (3.78) | .65 |
| Hand circumference (cm), mean (SD) | | 15.38 (1.61) | 14.8 (2.15) | 15.53 (1.77) | 15.37 (1.53) | 15.93 (1.67) | 14.86 (1.11) | 15.41 (1.14) | 16.48 (0.61) | .45 |
| **Stabilized time** | |  |  |  |  |  |  |  |  |  |
|  | After walking (seconds), mean (SD) | 357.06 (192.24) | 322.08 (167.1) | 397.18 (163.82) | 345.15 (283.71) | 358 (216.75) | 329 (128.29) | 306.5 (121.86) | 515 (156.46) | .55 |
|  | After running (seconds), mean (SD) | 790.67 (259.69) | 839.08 (298.93) | 833.55 (180.91) | 825.38 (233.81) | 514.5 (190.28) | 749.13 (163.72) | 809 (305.94) | 959.2 (314.83) | .05 |
